# An Algorithm for Protein Helix Assignment Using Helix Geometry

**DOI:** 10.1371/journal.pone.0129674

**Published:** 2015-07-01

**Authors:** Chen Cao, Shutan Xu, Lincong Wang

**Affiliations:** The College of Computer Science and Technology, Jilin University, Changchun, Jilin, China; Wake Forest University, UNITED STATES

## Abstract

Helices are one of the most common and were among the earliest recognized secondary structure elements in proteins. The assignment of helices in a protein underlies the analysis of its structure and function. Though the mathematical expression for a helical curve is simple, no previous assignment programs have used a genuine helical curve as a model for helix assignment. In this paper we present a two-step assignment algorithm. The first step searches for a series of bona fide helical curves each one best fits the coordinates of four successive backbone C_α_ atoms. The second step uses the best fit helical curves as input to make helix assignment. The application to the protein structures in the PDB (protein data bank) proves that the algorithm is able to assign accurately not only regular α-helix but also 3_10_ and π helices as well as their left-handed versions. One salient feature of the algorithm is that the assigned helices are structurally more uniform than those by the previous programs. The structural uniformity should be useful for protein structure classification and prediction while the accurate assignment of a helix to a particular type underlies structure-function relationship in proteins.

## 1 Introduction

Historically helices were proposed as the main secondary structure elements for proteins in 1951 [1] through model building using low-resolution X-ray diffraction data well before atomic coordinates could be determined from high-resolution data [2, 3]. As is evident from the helix model, the hydrogen bonding interaction between an amino (NH) group and a carbonyl (CO) group plays a decisive role in helix stability. The early recognition of the importance of hydrogen bonding interaction greatly affects our understanding of helices in proteins. In fact, the de-facto definitions of the three types of helices (*α*, 3_10_ and *π*-helices) are based on their distinct hydrogen bonding patterns: the hydrogen bonds between the CO of residue *i* and the NHs of residue *i* + 3, *i* + 4 and *i* + 5 are used respectively for the definitions of 3_10_, *α* and *π* helices. Largely as a consequence of the characteristic hydrogen bonding pattern and van der Waals repulsion, the backbone *ϕ* and *ψ* angles of a helix residue lie in two well-separated regions with the larger one corresponding to the right-handed helices while the much smaller one the left-handed ones. For the same reasons there exist no large variations in the derived geometrical restraints such as the virtual bond length between two successive C_*α*_ atoms, the virtual bond angle formed by a triple of successive C_*α*_ atoms and the dihedral angle formed by a quadruple of successive C_*α*_ atoms. The unique hydrogen bonding pattern and the small variation in the derived geometrical restraints provide the foundation for the previous assignment algorithms that use either hydrogen bond (dssp(Dictionary of Secondary Structure of Proteins) [4], stride [5] and secstr [6] and sst [7]) or geometrical restraint as inputs. The latter includes an early method developed by Levitt and Greer [8], define-s [9], p-sea [10], p-curve [11], palsse [12], stick [13], xtlsstr [14], kaksi [15] and the most recent program disicl [16]. The program stride [5] uses both hydrogen bond and geometrical restraint. At present, the hydrogen bond-based program dssp [4] is arguably the most popular helix assignment program. However, it has become clear quite early on that the hydrogen bonding pattern though unique for each helix type is not the sufficient condition for helix assignment, as is evidenced by the continuous development of restraint-based programs. Though there exist more than a dozen assignment programs at present, the accurate assignment of a protein helix remains to be a challenging problem [17, 18] as illustrated by the following comparative studies. It has been shown that the percentage of agreement between dssp, define-s and p-curve was only 63% on a residue basis [19]. The discrepancies and inconsistencies among the previous programs may well originate from their imprecise problem definitions since instead of rigorously following the helix geometry, they formulate the assignment problem as a restraint satisfaction problem in terms of the restraints that either could not be computed accurately (e.g. hydrogen bond) or have no precise range (e.g. *ϕ*/*ψ* angles) or are not sufficient for defining a general helical curve (e.g. virtual C_*α*_ bond length and angles).

In this paper we present a two-step algorithm that follows the division of the assignment problem into two separate problems: a minimization problem and a restraint satisfaction problem. The minimization problem is solved in the first step by a curve fitting algorithm that searches for a series of bona fide helical curves each one best fits the coordinates of a quadruple of successive C_*α*_ atoms. From the best fit helical curves we calculate three helix scores (one for each helix type), a helix axis angle and a C_*α*_ RMSD(root-mean-square deviation) for each residue that are in turn used in the second step as input to make helix assignment. A helix score for a residue quantifies the deviation from a standard protein helix (see section 2.2 for a precise definition of the term *the standard protein helix*) of a best fit helical curve that starts with the residue. The accurate assignment of a helix to a particular type is made possible by the following two observations: (1) each helix type has its own distinct helix score distribution and unique standard helix, and (2) a helix residue has a smaller helix score than a non-helix residue. The algorithm makes no use of hydrogen bond, *ϕ*/*ψ* angles, backbone NH or CO coordinate, virtual bond length or angle.

We have applied the assignment algorithm to identify the helices in the protein structures in the current version of PDB [20] and compared our assignment with those by the nine previous programs: dssp, stride, p-sea, kaksi, palsse, stick, xtlsstr, stt and disicl. The results demonstrate that the algorithm is able to assign accurately not only *α* but also 3_10_ and *π*-helices as well as left-handed helices [21]. To compare the structural distribution of the helices assigned by our and the previous programs, we have used a geometric clustering algorithm [22] to classify several sets of helices with the helices in each set all having the same length. The clustering analysis proves that the helices assigned by our algorithm are structurally more uniform than those by the previous ones. The accurately assigned helices and the helix clusters as well as the common structural features shared by all the helices in a cluster should be particularly useful for protein structure classification and prediction as well as secondary structure prediction while the accurate assignment of a helix to a particular type should provide a basis for the discovery of structure-function relationship in proteins.

The rest of the paper is organized as follows. In section 2 we describe the data set and present our algorithm. The application to the two sets of protein structures in the current PDB is detailed in section 3. In section 4 we compare the algorithm with the nine previous programs and discuss its key advantages. Finally we conclude the paper in section 5.

## 2 The data set and the algorithm

In this section we first describe the data set. We then present the helical curve fitting algorithm and the definitions and computations of helix score, helix axis angle and C_*α*_ RMSD as well as the definitions of three standard protein helices. Finally we detail the assignment algorithm itself.

### 2.1 The data set

To evaluate the performance of our algorithm, we have downloaded from the current version of the PDB a non-redundant set of x-ray structures (29,093 in total) with at most 70% sequence identity and each one has at least one helix according to the PDB. Out of them we have selected a set of 3,287 high-resolution structures (set 𝕊) each one has at least three helices, a resolution ≤ 2.0Å and a R-factor ≤ 25.0% to obtain the statistics for four helical parameters and two RMSDs. The set of the remaining 25,806 structures (set ℕ) is used to evaluate the performance of our algorithm and two previous programs dssp and stride. To compare our algorithm with the five programs (kaksi, plasse, stick, stlsstr and sst) that we are not able to obtain a local copy we have uploaded to a web server [23] a set of 100 x-ray protein structures (set 𝕋) with the first 50 structures having resolutions between 1.0Å–2.0Å and the rest having resolutions ≥ 2.5Å(1AKG,1BGF,1EZW,1GSU,1I1N,1K1B,1NTE,1O98,1PVM,1SJD,1UKF,1VZY,1XGW,1YQD,2ASC, 2BJI, 2CWH,2FD5,2GB2, 2GG6,2H1V,2I2C,2NSF, 2POK,2VBA,2W6K,2WRA,2X7H,2YSK,2ZJ3,3C9U,3EDF, 3GG7,3HG7,3IDV,3LCC,3LFJ,3P4H,3PUA, 3TOU,3V7Q,3ZOO;1AZ2,1BTN,1CAX,1F1F,1FXA,1HMY,1KX8, 1MJ9,1MSC,1NJ1,1PYP,1RIN,2CND,2GAE,2HAF,2HXB,2QQV, 2RJQ,2SPT,2WO7,2X2B,2Y5Q,2ZIY,3AT0, 3G0A,3IIA,3L1G,3M3T,3NPE,3O5K,3PGK,3PMQ,3QNT,3QYB,3T2Y,3UUF,3VGE, 3WMF,4AYH,4BOY, 4EWS,4GB0,4JVC,4LIF,4MBR,4N3G,4O1S,4PUT,4QGL,4TW8) to obtain their helix assignments. Set 𝕋 is also used for a comparison with the program disicl.

### 2.2 The curve fitting algorithm and helix assignment

Our solution to the helix assignment problem consists of two steps. The first step is to solve a minimization problem by a helical curve fitting algorithm that searches for a series of genuine helical curves each one best fits the coordinates of four successive C_*α*_ atoms. A helix model composed of a series of helical curves has been previously called a *polyhelix* [24]. We then define a standard protein helix for each helix type. The algorithmic solution to the minimization problem makes it possible to compute a helix axis angle *a*_*i*_, three helix scores *h*_*i*_, *g*_*i*_ and *π*_*i*_, and two C_*α*_ RMSDs for residue *i*. A helix score quantifies the deviation from a standard protein helix of the helical curve that begins with this particular residue. The score, axis angle and C_*α*_ RMSD are used in the second step as input to make helix assignment.

#### 2.2.1 The helical curve fitting algorithm

A general helical curve in three dimensional space could be represented as:

$$\begin{array}{c} \left[\begin{array}{c} x\\ y\\ z \end{array}]=[\begin{array}{c} x_{0}\\ y_{0}\\ z_{0} \end{array}]+R\;[\begin{array}{c} r\sin t\\ r\cos t\\ pt \end{array}\right] \end{array}$$ (1)

where **r** = {*x*, *y*, *z*} is a point on the curve, **r**_0_ = {*x*_0_, *y*_0_, *z*_0_} its origin, and **R** the rotation matrix that specifies its helical axis **n** with respect to a coordinate system. The first three helical parameters, radius (*r*), pitch (*p*) and turn angle (*t*), define a standard helical curve, *x* = *r* sin *t*, *y* = *r* cos *t*, *z* = *pt* with its origin at {1.0,0.0,0.0} and its axis along the +*Z* axis. Together with **n** and **r**_0_ these five parameters completely define a general helical curve. Though *r*, *p*, *t* could be computed directly from the virtual bond length, bond angle and dihedral angle of a quadruple of C_*α*_s [25], no simple analytic expression has been derived for the computation of a helical curve that best fits the coordinates of a quadruple of C_*α*_s, that is, a helical curve that has the minimum RMSD (Δ_*i*_) between the four C_*α*_s and their closest points on the curve. In fact, this minimization (or curve fitting) problem is equivalent to finding the solutions to a high-degree monomial. In the following we describe briefly an algorithmic solution to this minimization problem.

We begin with the computations of *r*, *p* and *t* using previously-derived analytic expressions [25], and denote their values as *r*_*m*_, *p*_*m*_ and *t*_*m*_. Then we proceed as follows to search discretely and exhaustively over two intervals, [*r*_*m*_ − *d*_*r*_, *r*_*m*_ + *d*_*r*_] and [*p*_*m*_ − *d*_*p*_, *p*_*m*_ + *d*_*p*_], for the *r* and *p* values of a helical curve such that it best fits the coordinates of a quadruple of C_*α*_s of residue *i*, *i* + 1, *i* + 2 and *i* + 3. Both *d*_*r*_ and *d*_*p*_ are user-specified constants.

1. Δ_*i*_ = ∞       {*the initial RMSD*}

2. For each *r* in [*r*_*m*_ − *d*_*r*_, *r*_*m*_ + *d*_*r*_]

  For each *p* in [*p*_*m*_ − *d*_*p*_, *p*_*m*_ + *d*_*p*_]

   Compute *t*   {*the turn angle*}

   Generate a helical curve   {*by* Eq 1}

   Best-fit the curve to the four C_*α*_ coordinates using singular-value decomposition(SVD) to compute **R**

   If *R*_*ms*_ < Δ_*i*_

    Δ_*i*_ = *R*_*ms*_

*r*_*i*_ = *r*, *p*_*i*_ = *p*, *t*_*i*_ = *t*, **R**_*i*_ = **R**

where *R*_*ms*_ is the RMSD between the quadruple of C_*α*_s and their closest points on the helical curve; *r*_*i*_, *p*_*i*_, *t*_*i*_ and **R**_*i*_ are, respectively, the helical parameters and rotation matrix for the helical curve that best-fits the quadruple. Given both *r* and *p* and the distance *d*_*i*,*i*+1_ between two consecutive C_*α*_s, *t* could be computed as follows: $t=2\;\text{arcsin}(0.5\sqrt{(d_{i,i+1}^{2}-p^{2})/r})$. Singular-value decomposition (SVD) is applied to compute Δ_*i*_ and rotation matrix **R**_*i*_; and from **R**_*i*_, the helical axis **n**_*i*_ for this helical curve could be calculated. In fact, the SVD step guarantees that the computed helical curve best fits the coordinates of the quadruple of C_*α*_s. A set of six helical parameters (*r*, *p*, *t*, Δ, **R** and **n**) is computed for a protein chain by sliding over its sequence a window of four C_*α*_ atoms.

#### 2.2.2 The computation of three helix scores and helix axis angle and C_*α*_ RMSD

Except for the last three residues at the C-terminus of a protein chain three helix scores, *h*_*i*_, *g*_*i*_ and *π*_*i*_, are computed for each residue *i*. They are used respectively for the assignment of *α*,3_10_ and *π* helices.

$$\begin{array}{c} \left\{h_{i},g_{i},\pi_{i}\right\}=\frac{{\left(r_{i}-\mu_{r}\right)}^{2}}{2\sigma_{r}^{2}}\times \frac{{\left(p_{i}-\mu_{p}\right)}^{2}}{2\sigma_{p}^{2}}\times \frac{{\left(t_{i}-\mu_{t}\right)}^{2}}{2\sigma_{t}^{2}}\times \frac{\Delta_{i}^{2}}{2\sigma_{{}_{\Delta }}^{2}} \end{array}$$ (2)

where *r*_*i*_, *p*_*i*_, *t*_*i*_, Δ_*i*_ are computed as above using a quadruple of C_*α*_s of residue *i*, *i* + 1, *i* + 2 and *i* + 3. The constants *μ*_*r*_, *σ*_*r*_; *μ*_*p*_, *σ*_*p*_; *μ*_*t*_, *σ*_*t*_ and *σ*_Δ_ are respectively the normal distribution parameters for *r*, *p*, *t*, Δ that are determined as follows over the respective data sets for *r*, *p*, *t*, Δ calculated on the non-redundant set 𝕊. We first apply the program dssp [4] to assign 31,383 *α*-helices, 11,926 3_10_-helices and 1,156 *π*-helices for the structures in 𝕊, and then for each helix type we calculate its *r*, *p*, *t* and Δ values. The *r*, *p*, *t* data sets for *π*-helix are calculated differently than those for either *α* or 3_10_-helices. If dssp assigns a *π*-helix say composed of residues *i*, *i* + 1, *i* + 2, *i* + 3, *i* + 4, then the final value for each *r*, *p*, *t* is the average over the three values for the first three residues, that is, $r=\frac{r_{i}+r_{i+1}+r_{i+2}}{3}$, $p=\frac{p_{i}+p_{i+1}+p_{i+2}}{3}$, $t=\frac{t_{i}+t_{i+1}+t_{i+2}}{3}$. Each triple of parameters *μ*_*r*_, *μ*_*p*_ and *μ*_*t*_ defines a standard helical curve for a helix type that represents an average over all the helices of that particular type in 𝕊. For ease of reference we call them respectively *the standard protein helix* for *α*,3_10_, and *π* helices. The helix scores *h*, *g* and *π* are computed with respect to the respective *α*,3_10_ and *π* standard protein helices. The score measures the local deviation of the helical curve from the standard protein helix for that particular helix type: the higher the score the larger deviation from the standard protein helix. The term $\frac{\Delta_{i}^{2}}{2\sigma_{\Delta }^{2}}$ quantifies the spatial difference between a C_*α*_ atom and its closest point on the helical curve. In fact, Δ together with the *r*, *p*, *t* terms in Eq 2 and the helical axis **n** provide a pure geometrical definition for a helix in a protein, that is, as long as a segment of C_*α*_ coordinates conform to a genuine helical curve, it is assigned as a helix.

The minimum RMSD Δ_*i*_ for residue *i* is computed over the quadruple of residues *i*, *i* + 1, *i* + 2 and *i* + 3 and thus is useful for the determination of the N-terminus of a helix. For the determination of the C-terminus of a helix, we have computed a C_*α*_ RMSD *δ*_*i*_ for residue *i* using up to four helical curves best fit to four successive quadruples of C_*α*_s starting with the quadruple of residues *i* − 2, *i* − 1, *i* and *i* + 1. The RMSD *δ* measures the goodness of fitting of up to four consecutive helical curves to seven successive C_*α*_ atoms. In the current implementation *δ* is used for the extension of the C-terminus of a 3_10_-helix and the possible merge of two adjacent *α*-helices. In addition to the helix scores and *δ*, we have also calculated the angle between two successive helical axes **n**_*i*−1_ and **n**_*i*_ and use it as input to the assignment algorithm. This angle measures the bending of the current helical curve starting at residue *i* relative to the previous helical curve starting at residue *i* − 1. For ease of reference we call this angle *helix axis angle* and call the four variables *r*_*i*_, *p*_*i*_, *t*_*i*_, *a*_*i*_ *the four helical parameters* for residue *i*. For the first residue at the C-terminus we set *a*_*i*_ = 0.0° and for a genuine helical curve all of its *a*_*i*_s are zero. The set of four helical parameters for all the quadruples of consecutive C_*α*_s in a protein chain are computed by sliding over its sequence a window of four C_*α*_ atoms.

Four thresholds *h*_*T*_, *h*_*max*_, *g*_*T*_ and *π*_*T*_ for the three helix scores, four thresholds *a*_*T*_, *a*_*max*_, *a*_*G*_ and *a*_*I*_ for the helix axis angle and two thresholds, *δ*_*G*_ and *δ*_*max*_, for *δ* are required by our assignment algorithm. These ten thresholds are determined as detailed late by the analyses of the statistics for both helical parameters and RMSDs obtained on all the helices in 𝕊 assigned by the program dssp.

The five parameters, *r*, *p*, *t*, **r**_0_, **n**, completely defines a right-handed helical curve. By inverting just one component of every C_*α*_ coordinate, say from {*x*, *y*, *z*} to {−*x*, *y*, *z*}, the same five parameters together with the axis angle and two RMSDs could be computed similarly and used for the assignment of the left-handed helices.

#### 2.2.3 The helix assignment algorithm

The assignment proceeds as follows using helix score, axis angle and C_*α*_ RMSD as well as the ten thresholds as input. The assignment for each protein chain starts with *π*-helix.

let *b*_*i*_ = 0, *i* = 0, …, *n*

while *i* < *n* AND *b*_*i*_ == 0  {*residue i has NOT been assigned*}

If *π*_*i*_ < *π*_*T*_ AND *a*_*i*_ < *a*_*I*_ AND *a*_*i*+1_ < *a*_*I*_ AND *a*_*i*+2_ < *a*_*I*_ {*the N-terminus of a π helix*}

*i* ∈ *π*-helix  {*assign residue i to π helix*}

*b*_*i*_ = 1

*i* + +

 while *π*_*i*_ < *π*_*T*_ AND *i* < (*n* − 3) AND *b*_*i*_ == 0

*i* ∈ *π*-helix

*b*_*i*_ = 1

*i* + +

for *j* = *i*, *i* + 1, *i* + 2, *i* + 3

*j* ∈ *π*-helix

*j* + +

*b*_*j*_ = 1

*i* = *j*

*i* + +

where *n* is the number of residues in a protein chain, *i* residue index, and *π*_*T*_ a threshold for helix score *π* and *a*_*I*_ a threshold for helix axis angle used only in *π*-helix assignment.

Next the algorithm assigns 3_10_-helices for the remaining residues of the same protein chain.

while *i* < *n* AND *b*_*i*_ == 0  {*residue i has NOT been assigned*}

 If *g*_*i*_ < *g*_*T*_ AND *a*_*i*_ < *a*_*G*_  {*the N-terminus of a* 3_10_ *helix*}

*i* ∈ 3_10_-helix  {*assign residue i to* 3_10_ *helix*}

*b*_*i*_ = 1

*i* + +

while *g*_*i*_ < *g*_*T*_ AND *i* < *n* AND *b*_*i*_ == 0

*i* ∈ 3_10_-helix

*b*_*i*_ = 1

*i* + +

if *δ*_*i*_ < *δ*_*G*_ AND *δ*_*i*+1_ < *δ*_*G*_  {*assign two more residues to* 3_10_ *helix*}

*i* ∈ 3_10_-helix

*b*_*i*_ = 1

*i* + +

*i* ∈ 3_10_-helix

*b*_*i*_ = 1

*i* + +

where *g*_*T*_, *a*_*G*_ and *δ*_*G*_ are respectively the thresholds for helix score *g*_*i*_, axis angle and RMSD *δ* used only in 3_10_-helix assignment.

Finally the algorithm assigns *α*-helices for the remaining residues of the same protein chain.

while *i* < *n* AND *b*_*i*_ == 0  {*residue i has NOT been assigned*}

 If *h*_*i*_ < *h*_*T*_ AND *a*_*i*_ < *a*_*T*_ AND *h*_*i*+1_ < *h*_*T*_  {*the N-terminus of a helix*}

*i* ∈ *α*-helix  {*assign residue i to a helix*}

*b*_*i*_ = 1

*i* + +

while *h*_*i*_ < *h*_*T*_ AND *i* < *n* AND *b*_*i*_ == 0

*i* ∈ *α*-helix

*b*_*i*_ = 1

*i* + +

*i* + +

where *h*_*T*_ a threshold for helix score *h*_*i*_. For the assignment of *α*-helices only, additional steps may be needed to merge two *α*-helices adjacent in protein sequence and to extend their C-termini. The merge of two adjacent *α*-helices proceeds as follows. For every pair of adjacent helices less than four residues apart, if the axis angle *a*_*i*_ ≤ *a*_*max*_, helix score *h*_*i*_ < *h*_*max*_ and *δ*_*i*_ < *δ*_*max*_ for every intermediate residue *i*, then the two helices are merged into a single helix. The thresholds *h*_*max*_, *a*_*max*_ and *δ*_*max*_ are the respective thresholds for axis angle, helix score and *δ*. The threshold *δ*_*max*_ is used only in the merge step.

After the merge step the C-terminus of an *α*-helix may be extended as follows.

let *j* − 1 be the last residue of an *α*-helix assigned above

if *j* < *n* AND *b*_*j*_ == 0  {*residue *j* has NOT been assigned*}

  If *h*_*j*_ < *h*_*max*_ AND *a*_*j*_ < *a*_*max*_

*b*_*j*_ = 1

*j* ∈ *α*-helix

   j + +

*b*_*j*_ = 1

*j* ∈ *α*-helix

where *h*_*max*_ and *a*_*max*_ are the same thresholds in the merge step.

Though the three types of helices are assigned similarly at individual helix level, no merge step is necessary for either 3_10_ or *π* helix since there exist rarely 3_10_ or *π* helices with more than eight residues. In our current implementation, no extension is made for *π* helix.

A left handed helix is assigned similarly except that the helical parameters and RMSDs used in Eq 2 are those computed from a quadruple of inverted C_*α*_ coordinates as described above.

### 2.3 Helix classification

To better compare the assigned helices made by our and previous programs and to characterize their structures we have classified them using our geometric clustering algorithm [22]. The clustering is performed on sets of helices that have the same length. The RMSD threshold for clustering is 1.5Å.

## 3 Results

The input to our algorithm are the helix scores *h*_*i*_, *g*_*i*_, *π*_*i*_, axis angle *a*_*i*_ and *δ*_*i*_ for every residue *i* and their corresponding thresholds *h*_*T*_, *h*_*max*_, *g*_*T*_, *π*_*T*_, *a*_*T*_, *a*_*max*_, *a*_*G*_, *a*_*I*_, *δ*_*G*_ and *δ*_*max*_. The computations of *h*_*i*_, *g*_*i*_, *π*_*i*_ by Eq (2) require the seven parameters *μ*_*r*_, *σ*_*r*_;*μ*_*p*_, *σ*_*p*_;*μ*_*t*_, *σ*_*t*_ and *σ*_Δ_ to be known in advance. In this section we begin with the determination of these seven parameters and the ten thresholds through the statistical analyses of the data sets for *r*, *p*, *t*, Δ, *a* and *δ*. These data sets are computed using the helical curves fitted to the dssp-assigned helices in set 𝕊. We then present and compare the assignments made for both ℕ and 𝕋 by our algorithm and nine previous programs including three hydrogen-bond based ones (dssp (version 2.2.1), stride and stt) and six geometrical restraint based-ones (p-sea, palsse, stick, xtlsstr, kaksi and disicl). Finally we describe briefly the algorithmic implementation.

### 3.1 The statistical analyses of helical parameters and RMSDs

The premises of our assignment algorithm are (1) each helix type has its own distinct helix score distribution and (2) a helix residue has a smaller helix score than a non-helix residue. To test how good the premises are we first use the program dssp to divide all the protein residues in 𝕊 into five groups: *α*-helix (H), 3_10_-helix (G), *π*-helix (I), *β*-sheet or extended configuration (E) and the other (R), and then for each group obtain separate statistics for the four helical parameters and two RMSDs. The statistics for different helix types are computed somewhat differently. The *α*-helix statistics include the data for every residue of an *α*-helix except the last three residues of its C-terminus. The dssp assigned 3_10_-helices (group G) have ≥ 3 residues per helix with an average length of 3.3 residues. The statistics for the 3_10_-helices are computed over 1–3 sliding windows starting with the residues *i* − 1, *i*, *i* + 1, *i* + 2 where *i* is the first residue of a 3_10_-helix. The four helical parameters and two RMSDs for the dssp assigned *π*-helices (group I) have larger variations than those for either *α* or 3_10_ helices. However, we found that the *r*, *p*, *t* values averaged over the first three residues of every dssp assigned *π*-helix have well-defined distributions. Consequently the *π*-helix statistics is obtained over such averages.

**Fig 1:**
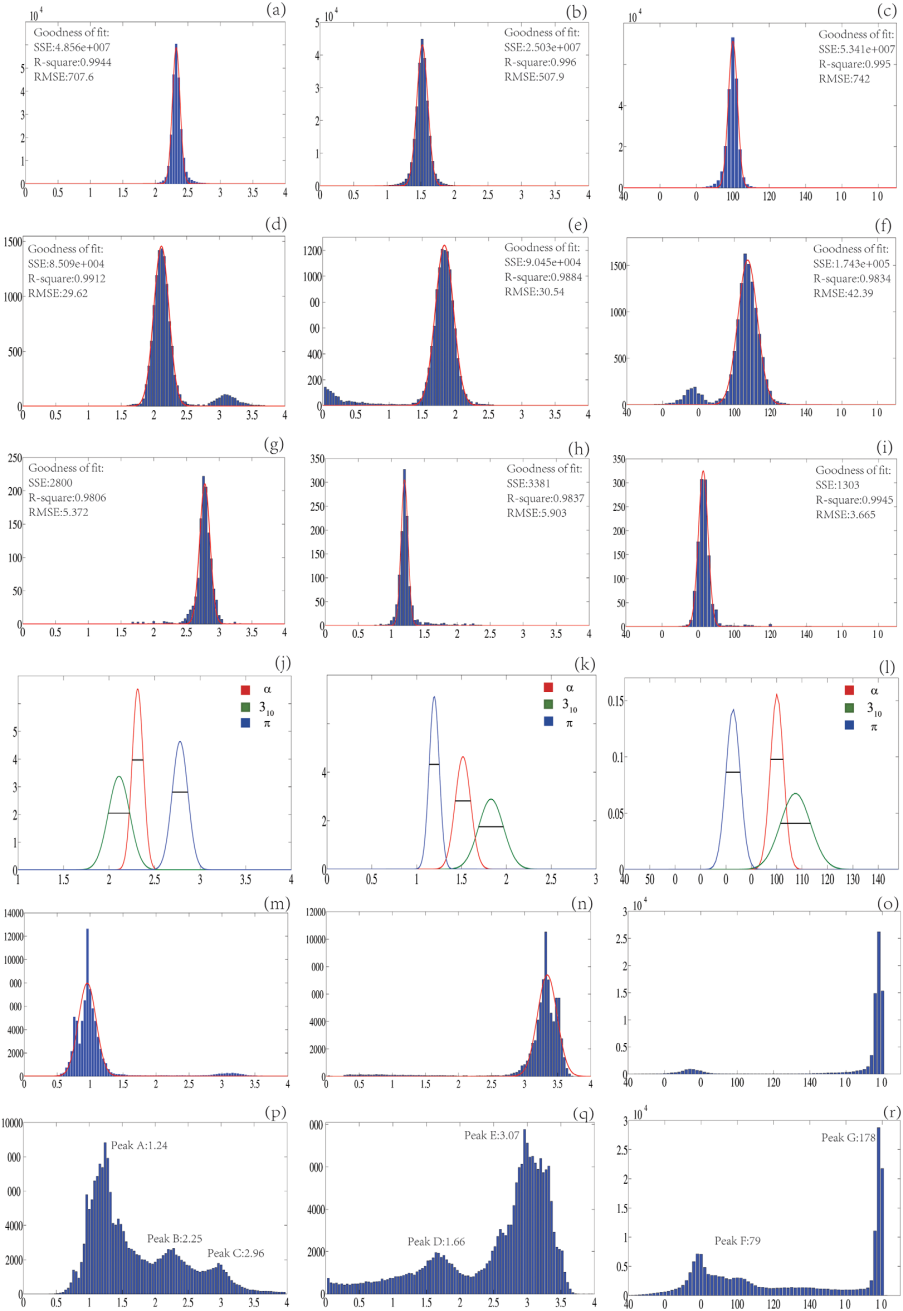
The *r*, *p*, *t* statistics for five different groups (H, G, I, E, R). The five triples of figures (**a**, **b**, **c**), (**d**, **e**, **f**), (**g**, **h**, **i**), (**m**, **n**, **o**) and (**p**, **q**, **r**) are respectively the *r*, *p*, *t* distributions for *α*-helix (H), *π*-helix(G), 3_10_-helix (I), *β*-sheet (E) and the other (R) groups. Except for figures **j**, **k** and **l** whose y-axes are the scale for a normal distribution, the y-axes in all the other figures are the number of residues. For each row, the x-axis is respectively the radius (*r* in Å), pitch (*p* in Å) and turn angle (*t* in degree). There is a small peak in figures **d**, **e**, **f** from the left-handed helices. Figures **j**, **k** and **l** display the fitted *r*, *p*, *t* normal distribution curves for the three helix types where each line segment has a length of 2.0*σ* where *σ* is the standard deviation. All the overlaps are well below the 2*σ* lines except for *t* statistics where the overlap between the *α*-helix normal curve and 3_10_-helix normal curve is slightly above the 2*σ* line.

The statistics for the four helical parameters and two RMSDs are fitted to a normal distribution to compute their normal distribution parameters *μ* and *σ*. As shown in Table 1 and Fig 1, for every helix type each *r*, *p*, *t* statistics (Fig 1a–1i) could be fitted very well to a normal probability function. In addition, the *σ*s are rather small and the *μ*s well separated from each other (*δ*_*p*_ in Table 1 and Fig 1j, 1k and 1l). More importantly the *r*, *p*, *t* statistics for a helix group (H or G or I) are well separated from the statistics for group E (Fig 1m, 1n and 1o). Furthermore, the largest peaks in the *r*, *p*, *t* distributions for a helix group are well separated from the largest peaks in the *r*, *p*, *t* distributions for group R (Fig 1p, 1q and 1r) though the largest peaks in the former do overlap with the minor peaks in the latter. The statistics for *a*, Δ and *δ* for each helix type could also be fitted to a normal probability function reasonably well (data not shown). The *μ* and *σ*s for Δ are respectively (0.02,0.031), (0.045,0.038) and (0.129,0.056) for *α*, 3_10_ and *π* helices. The smallness of the *σ*s, the large separation between the *μ*s for different helix types, and the large difference between the *μ*s for a helix group and the *μ*s for either E or R group are consistent with the two premises. They are the reasons why the helix scores *h*, *g*, *π* could be used to assign a helix to a particular type.

**Table 1:** The normal distribution parameters (*μ*, *σ*) for *r*, *p*, *t*. The residues have been divided into five groups (H, G, I, E, R) based on the secondary structure elements assigned by dssp. The last row (*δ*_*P*_) shows the difference in *μ* between H and the other four groups. The unit for both *r*, *p* is Å while *t* is in degree.

	*α*-helix (H)			3_10_-helix (G)			*π*-helix (I)			*β*-sheet (E)			the other (R)		
	*r*	*p*	*t*	*r*	*p*	*t*	*r*	*p*	*t*	*r*	*p*	*t*	*r*	*p*	*t*
*μ*	2.314	1.516	100.1	2.109	1.829	107.4	2.779	1.196	82.8	0.96	3.34	177.9	1.26	3.04	178.9
*σ*	0.061	0.086	2.56	0.118	0.138	5.90	0.086	0.056	2.80	0.146	0.158	2.12	0.218	0.311	1.78
*δ*_*P*_	0.0	0.0	0.0	0.203	0.313	7.35	0.465	0.32	17.24	1.354	1.824	77.8	1.054	1.524	78.8

**Fig 2:**
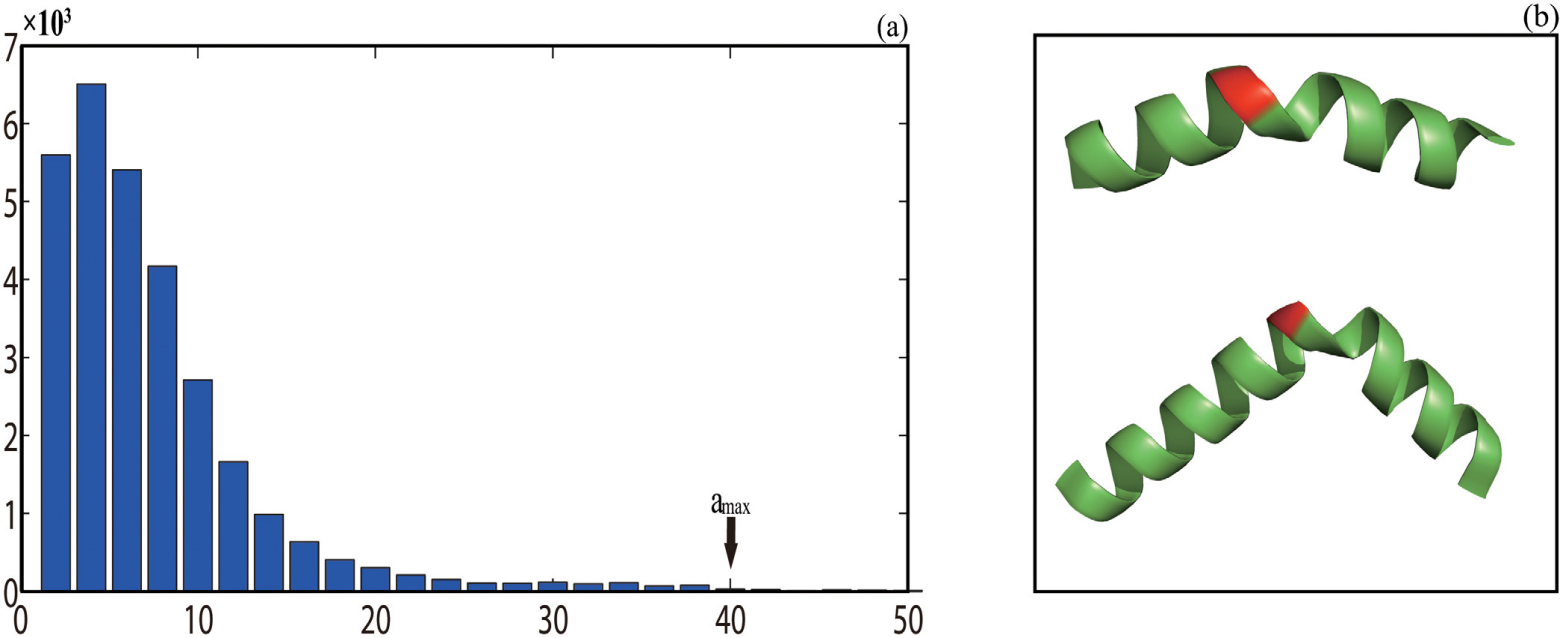
A histogram of helix axis angle *a* (a) and the threshold *a*_*max*_ (b). The x-axis in (a) is the angle in degree while the y-axis is the number of residues. In (b) the axis angle of residue 28 (colored in red and located in the middle of a segment, the top figure) in a protein (pdbid 4CXF) has *a*_28_ = 37.72° while the angle of residue 53 (colored in red and located in the middle of a segment, the bottom figure) in a protein (pdbid 1SQG) has *a*_53_ = 44.95°. With a threshold of *a*_*max*_ = 40.0°, the first segment is assigned by our algorithm as a single *α*-helix while the second segment is divided into two different helices. In contrast both segments are assigned as a single helix by dssp.

From the statistics for the helix scores *h*, *g*, *π* obtained on all the helices in 𝕊 assigned by the program dssp we set *h*_*T*_ = 20.0, *g*_*T*_ = 6.0, *π*_*T*_ = 14.0. They are used respectively in *α*,3_10_ and *π* helix assignment. Similarly from the statistics for axis angle *a* obtained on the same set 𝕊 we set *a*_*T*_ = 20.0°, *a*_*G*_ = 10.0°, *a*_*I*_ = 20.0°. They are also used respectively in *α*,3_10_ and *π* helix assignment. From the distributions shown in Table 2 and Fig 2, the default value for the threshold *a*_*max*_ (Fig 2b) is set to 40.0°, a value that is well beyond the distribution for *a* in the set of 382 ultra-high resolution structure each one has a resolution ≤ 1.0Å. A statistical analysis of the axis angle on all the helices assigned by the program dssp on set 𝕊 shows that more than 83% of the C-terminal residues of the helices have the axis angle less than 40.0° (S1 Fig) so the same threshold is used for the termination of *α*-helix assignment. The default value for threshold *h*_*max*_ is set to 160.0, well above *h*_*T*_ = 20.0 used for *α*-helix assignment because a merge step is only triggered for the part of helix that has large local deviations from a genuine helical curve. The thresholds *a*_*max*_, *h*_*max*_ and *δ*_*max*_ decide whether two adjacent helices should be merged. We set *δ*_*max*_ = 0.3 based on a statistical analysis of the *δ* distribution on the same set 𝕊. The threshold *δ*_*G*_ that plays an important role in how far the C-terminus of a 3_10_-helix is to be extended is set to 0.12, based on the statistics that ≤ 2.0% *α*-helices have a *δ* value larger than 0.12.

**Table 2:** The statistics of *a*, Δ and *δ*. The data are obtained from a set of the dssp assigned *α*-helices on a set of 382 ultra-high resolution (≤ 1.0Å) x-ray structures. Each column presents respectively a data range and the number of residues inside that range. Both RMSDs, Δ and *δ*, have unit Å while *a* is in degree.

Parameter	range, residue	range, residue	range, residue	range, residue
*a*	0.0–6.0, 6711	6.0–12.0, 1198	12.0–24.0, 273	≥ **40.0, 12**
Δ	0.0–0.06, 7109	0.06–0.12, 972	0.12–0.24, 176	≥ 0.24, 5
*δ*	0.0–0.06, 7101	0.06–0.12, 437	0.12–0.24, 131	≥ **0.30, 501**

### 3.2 The helix assignment

We have applied our algorithm as well as the dssp and stride programs to assign *α*, 3_10_ and *π* helices for the 25,806 protein structures in ℕ. For each helix type, the total numbers of helices and residues assigned by each program are listed in Table 3. On set ℕ our algorithm assigns 178,104 *α*-helices with a length from 4 to 212 residues and the most frequently appeared helix has a length of 10 residues; 70,208 3_10_-helices with a length from 3 to 14 residues with the most frequently appeared ones has a length of 3 residues; and finally 6,600 *π*-helices with a length from 5 to 18 residues and the most frequently appeared ones has a length of 5 residues. Due to the difficulty of obtaining a local copy for the programs palsse, stick, xtlsstr, kaksi and sst we have tested them on the set 𝕋 of 100 selected x-ray structures by uploading each one to a web server [23] to obtain their assignments (Table 4). The comparison with the program disicl is made on the same set 𝕋. We are not able to compare our algorithm with the rest of the published assignment programs because we could not get a working copy for any of them.

**Table 3:** Helix assignment on a residue and a helix basis by our algorithm, dssp and stride. The third column for each helix type presents respectively the range in helix length and the length of the most frequently appeared helices.

Program	*α*-helix			3_10_-helix			*π*-helix		
	helix	residue	range,length	helix	residue	range,length	helix	residue	range,length
dssp	187,594	2,122,751	4–164, 4	68,093	228,690	3–17, 3	6,579	35,306	5–15, 5
stride	176,217	2,183,629	4–281, 10	70,974	228,324	3–16, 3	205	1,037	5–10, 5
our	178,014	1,984,067	4–212, 10	70,208	239,783	3–14, 3	6,600	36,960	5–18, 5

**Table 4:** The assignments on 𝕋 by our algorithm and the nine previous programs. The assignments are made for a set of 100 x-ray structures with different resolutions. The first row is the total number of residues. All the other rows are the agreement between a pair of programs in percentage. The percentage is computed as $\frac{n}{n_{1}+n+n_{2}}$ where *n* is the number of residues assigned by both programs while *n*_1_ and *n*_2_ are respectively the numbers of residues assigned only by the first and second programs.

Program	Our	dssp	stride	p-sea	kaksi	plasse	stick	xtlsstr	sst	disicl
Total	8670	9052	9392	8282	9379	12332	8309	9704	8994	8432
**Our**		**89.2**	**86.8**	**81.5**	**76.1**	**67.9**	**76.2**	**78.4**	**78.3**	**77.7**
dssp			*90.6*	*80.7*	*76.4*	*71.2*	*75.5*	*81.4*	*77.4*	*76.8*
stride				83.7	79.9	73.9	78.4	83.1	80.3	79.5
p-sea					79.7	66.8	82.0	76.1	81.5	74.3
kaksi						74.9	76.6	77.7	79.0	74.5
plasse							66.5	74.1	71.1	61.4
stick								72.3	77.4	69.3
xtlsstr									77.0	72.8
sst										71.2

### 3.3 The algorithmic implementation

We have implemented our helix assignment algorithm in C++ and included it as a module in our structure analysis and visualization program written in C++/Qt/openGL. The default values for the two parameters, *δ*_*r*_ and *δ*_*p*_, required for the computations of the helix parameters *r*, *p* are set to 0.25 and the step size for both intervals, [*r*_*m*_ − *δ*_*r*_, *r*_*m*_ + *δ*_*r*_] and [*p*_*m*_ − *δ*_*p*_, *p*_*m*_ + *δ*_*p*_], is 0.01. The program is available upon the request.

## 4 Discussion

In this paper we have developed a two-step algorithm that follows the division of the assignment problem into two sub-problems: a minimization problem and a restraint satisfaction problem. The first step solves the minimization problem by a newly-developed helical curve fitting algorithm. From the best fit helical curves we then calculate helix score, axis angle and C_*α*_ RMSD for each residue that are in turn used as restraints for helix assignment. In the following we first discuss the rational and motivation behind the two-step algorithm and the two premises of the algorithm. Then we discuss the advantages of our algorithm over the previous programs through the detailed comparisons of the assignments made for both ℕ and 𝕋, and through structure classification of the assigned helices. Finally we illustrate the biological significance of the assignment of a helix to a particular type by showing a correlation between the *π*-helices assigned by our algorithm and protein-ligand binding sites.

### 4.1 A two-step solution to the helix assignment problem and the two premises

As has been well-documented before [18, 19] that there exist large discrepancies (up to 37%) among the previous helix assignment programs. These programs share two features: (1) none of them use a genuine helical curve as a model, and (2) they provide a one-step solution to the assignment problem formulated as a restraint satisfaction problem where the restraints could be either the hydrogen bond between backbone atoms or the geometrical restraints such as backbone *ϕ*/*ψ* angles, virtual C_*α*_ bond length and angle. Because of the error in the computed hydrogen bond energy or the variations in the geometrical restraints, the programs that use different types of restraints are expected to generate different assignments for the same protein (see Table 4 for examples). In contrast, we divide the assignment problem into two sub-problems with the first being a minimization problem. The algorithmic solution to the minimization problem generates a series of best fit helical curves that are in turn used as input to the assignment algorithm. The accurate computation of these helical curves leads to the high accuracy and consistency since unlike the previous geometrical restraint-based programs the strict requirement that all the helices must fit reasonably well to a helical curve greatly reduces the possible variation in the input restraints and that in turn leads to the structural uniformity of the assigned helices.

The statistical analyses of the helical parameters *r*, *p*, *t* for all the residues in 𝕊 show that in general their distributions for different types of secondary structure elements have almost no overlap (Fig 1j–1l). In addition, for each helix type the axis angle *a* and the RMSDs Δ, *δ* are usually rather small (Table 2). These observations (computational results) are consistent with the two premises of our assignment algorithm. They are the reasons why our algorithm could not only assign a backbone segment to a helix but also output its helix type.

### 4.2 The comparisons with the previous programs in helix assignment

In this section we compare the helix assignments by our algorithm, dssp and stride on ℕ (25,806 structures) as well as the assignments by our algorithm and nine previous programs on 𝕋 (100 structures). We also illustrate the differences in assignment by examples.

**Fig 3:**
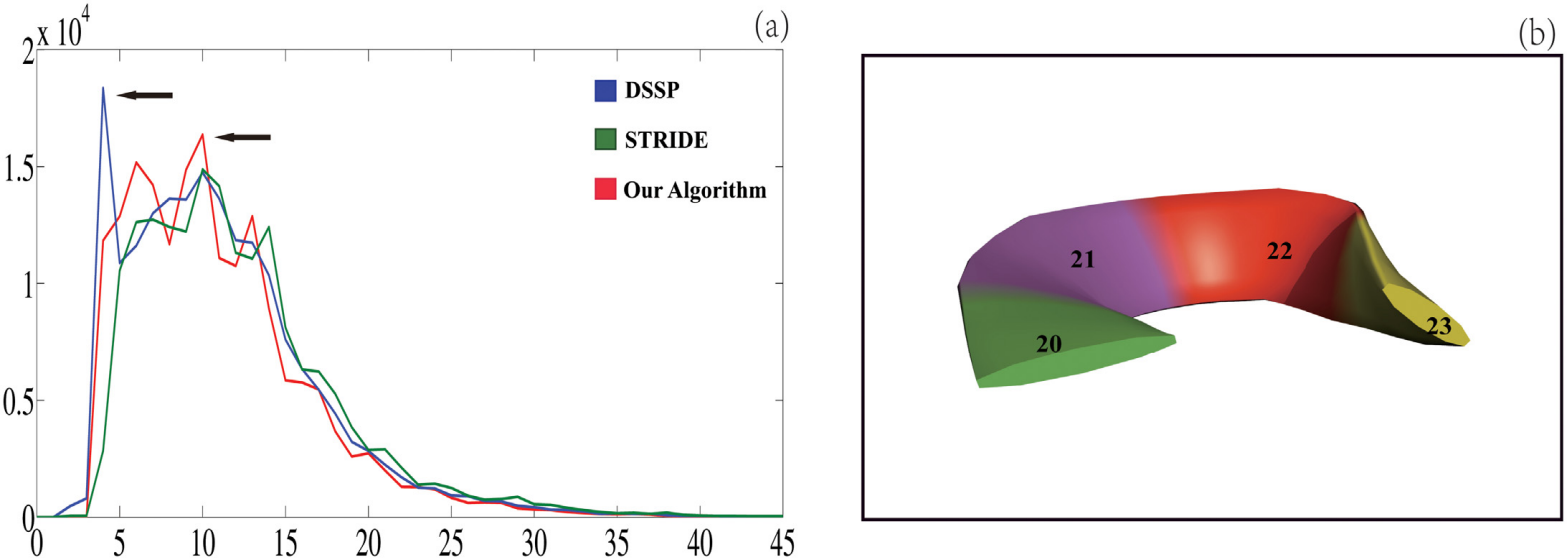
The distributions of the lengths of the *α*-helices from our algorithm, dssp and stride (a), and an example of 4-residue *α*-helix by dssp(b). The x-axis in (a) is the helix length while the y-axis is the number of the helices with that particular length. The two arrows point to the most frequently appeared helices assigned by dssp and by both our algorithm and stride. The right figure (b) depicts a dssp-assigned 4-residue *α*-helix in a protein (pdbid 1CC5) that is not assigned to a helix by our algorithm.

For the *α*-helices in ℕ, our assignment agrees very well with both dssp and stride (Table 5). Specifically the agreement with dssp is 95.3% on a residue basis and 95.1% on a helix basis, and with stride 95.4% and 90.4%. In addition the most frequently appeared helices from our algorithm and stride have the same length (10 residues) (Fig 3a). However, the most frequently appeared helices by dssp have a length of only 4 residues. This large discrepancy may originate from the fact that dssp relies only on two *i*, *i* + 4 hydrogen bonds for assigning a 4-residue helix but it is likely that two such bonds are not strong enough to constraint the C_*α*_s to a helical curve and thus our algorithm does not assign them as a helix (Fig 3b). In addition to hydrogen bond, stride also uses backbone *ϕ*/*ψ* angles as restraint to exclude such 4-residue helices. In fact it seems that the criteria used by stride are so restrictive that it excludes most of the 4-residue helices assigned by both our algorithm and dssp (Fig 3a). Though the total numbers of 3_10_-helices from the three programs, 68,093 (dssp), 70,974 (stride) and 70,208 (our algorithm), are rather similar (Table 3), there exist obvious differences at both residue and helix levels with the best agreement between our algorithm and either dssp or stride to be ≤ 60%. The discrepancies suggest that the few hydrogen bonds in a 3_10_-helix whose average length is only 3.3 residues are not strong enough to constraint the C_*α*_s to a helical curve. The same reason may explain the ≥ 25% difference in *π*-helix assignment between our algorithm and dssp since the most frequently appeared *π*-helices have a length of only 5 residues. For reasons unknown to us, stride fails to assign most *π*-helices: the agreement between our algorithm and stride is only 2.9%. The discrepancies also suggest that especially for 3_10_ and *π*-helices the existence of the characteristic hydrogen bonding pattern between protein backbone atoms used by the hydrogen bond-based programs is not a sufficient condition for the backbone segment to fit well to a helical curve.

**Table 5:** The comparisons of helix assignment on ℕ by our algorithm, dssp and stride. The third column (in boldface) is the agreement on a residue basis computed as $100.0\times \frac{n}{N}$ where *N* is the total number of residues assigned by our program, and *n* the number of the residues assigned by both our algorithm and either dssp or stride. The agreement on a residue basis on *π*-helix assignment between our algorithm and stride is very poor, only 2.9%. The 4th-10th columns present various agreements (disagreements) on a helix basis. The columns A, B, C show respectively the agreement with at most one residue difference, with the exclusion of the N-terminal residue and the exclusion of the C-terminal residue. The column A+B+C (in boldface) sums up the agreements on a helix basis. The next two columns D and E present respectively the helices assigned only by our algorithm and the helices assigned only by either dssp or stride. The last column F shows the set of helices each one has been assigned by either dssp or stride as a single helix but is divided into at least two helices by our algorithm (see Figs 4 and 5). All the data are in percentage.

Program	helix	residue(%)	A (%)	B (%)	C (%)	A+B+C(%)	D(%)	E(%)	F(%)
dssp	*α*	**95.3**	73.3	9.4	12.6	**95.3**	1.3	8.2	1.9
	3_10_	**56.9**	55.4	2.2	2.5	**60.1**	39.9	33.0	0.01
	*π*	**74.1**	74.9	2.5	1.6	**79**	21.1	20.5	0.01
stride	*α*	**95.4**	65.2	10.7	14.8	**90.7**	2.1	4.0	3.7
	3_10_	**57.0**	55.0	2.3	2.2	**61.5**	40.6	42.6	0.1

In addition to set ℕ, a set 𝕋 of 100 protein structures with different resolutions have been selected for the comparisons with nine previous programs including both hydrogen bond-based and geometrical restraint-based. Except for the four programs dssp, stride, p-sea and disicl we are not able to get a local copy for the other five programs. Since we could only make p-sea to work on less than a half of the total structures in ℕ we only present its assignment on 𝕋. The comparison with the program disicl is made only on 𝕋. As shown in Table 4, our algorithm agrees with either dssp or stride better than the other programs do. The agreements between any pair of the six geometrical restraint-based programs (p-sea, kaksi, plasse, stick, xtlsstr and disicl) are between 61.4% and 82.0% and their agreements with the three hydrogen bond-based programs (dssp, stride and sst) are between 71.2% and 83.7%. The most recent geometrical restraint-based program disicl developed mainly for the analyses of the trajectories from molecular dynamics simulations performs below the average as judged by its assignment agreements with dssp (76.8%), our algorithm (77.7%) and the seven other programs (61.4%–79.5%). Our algorithm tends to assign a lower number of helices than the previous programs do likely because of the strict requirement that for a backbone segment to be a helix it must fit to a genuine helical curve. Compared with the other geometrical restraint-based programs, the helices assigned by our algorithm tend to be longer likely because of the merge step taken by our algorithm. For the comparisons on 𝕋 we do not separate the assigned helices into different types because the helix assignment downloaded from the web server does not have type information. In summary, if we use the assignment made by the program dssp as the standard, our algorithm is the most accurate among the six geometrical restraint-based programs with them we have compared.

**Fig 4:**
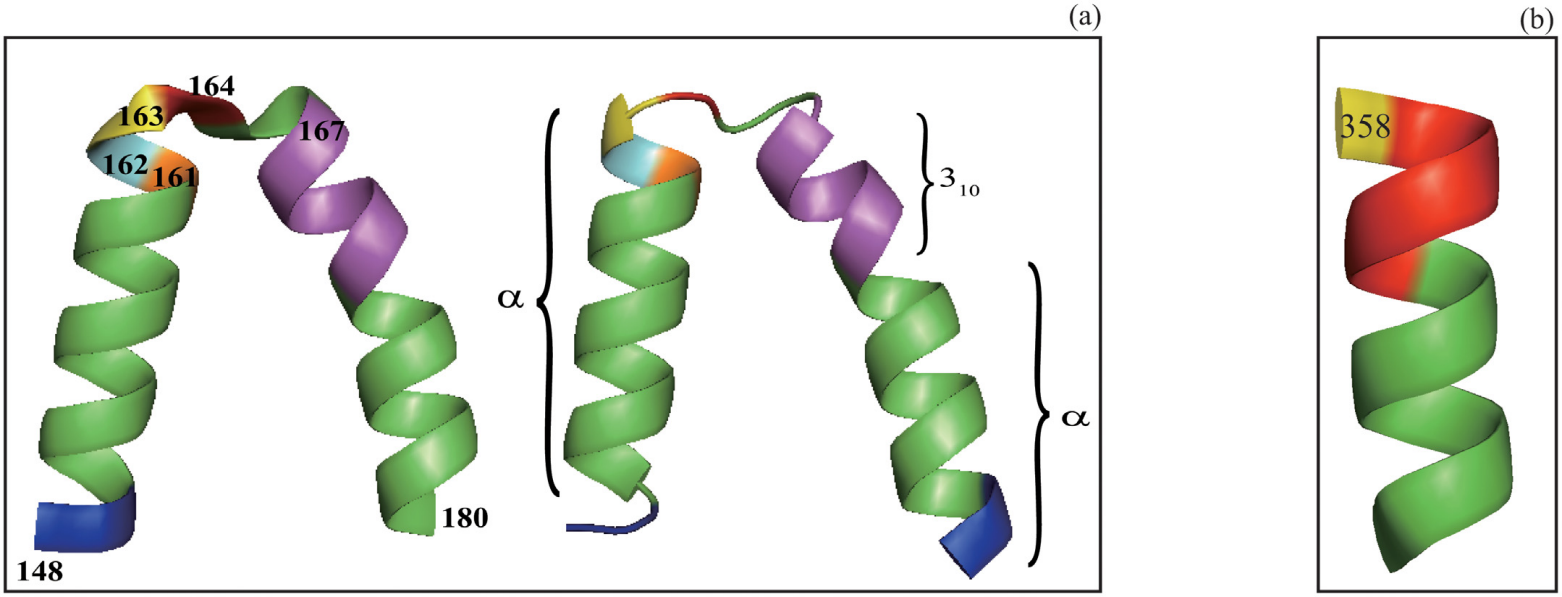
The illustration of the differences in assignment by our algorithm and dssp. In (a) a segment (residues 148–180) in a protein (pdbid 1DI1) assigned as a single *α*-helix by dssp is shown in the left with residue 163 colored in yellow. Our algorithm divides the same segment into three different helices (the right figure): *α*-helix–3_10_–helix–*α*-helix. The first helix assigned by our algorithm starts with residue 150 since both residues 148 and 149 have *h*_*i*_ > *h*_*T*_ and stops at residue 163 (colored in yellow) since its *h*_*i*_ > *h*_*T*_ and no merge is triggered because that *a*_*i*_ = 85.12° > *a*_*max*_ = 40.0° for the three residues 164 (colored in red), 165 and 166. The residues 164–166 all have *h*_*i*_ > *h*_*T*_, so the next helix starts with residue 167. In contrast to dssp our algorithm assigns the segment of residues 167–171 as a 3_10_contentsline helix (colored in purple), and assigns the remaining residue 172–182 as an *α*-helix. Compared with dssp, the C-terminus has been extended by two residues (A181 and Q182, colored in blue). In (b) dssp divides the segment (347–358) in a protein (pdbid 3I32) into two helices: *α*-helix (347–353, green) and *π*-helix (354–358, red) based on the strength of hydrogen bonding interaction. In contrast, our algorithm assigns the whole segment (347–357) as a single *α*-helix but excludes the last residue 358.

**Fig 5:**
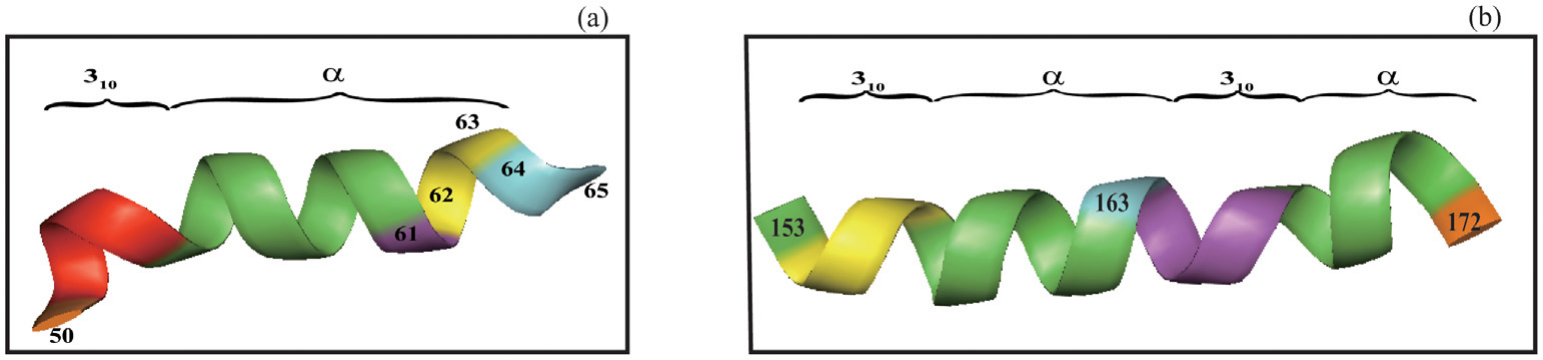
The illustration of the differences in assignment by our algorithm and dssp. In (a) dssp assigns the entire segment (51–65, excluding P50) in a protein (pdbid 3OY9) as an *α*-helix. Our algorithm divides it into two helices: 3_10_-helix (51–52, red) and *α*-helix (53–63, green, purple, yellow). The *α*-helix stops at N63 since the C_*α*_ RMSD *δ* values for residue 64 and 65 are respectively 0.541, 0.431, none of them less than *d*_*max*_ = 0.3. In contrast, the dssp assigned helix extends to residue 65. However, as shown in the left figure, the C_*α*_ coordinates of both residue 64 and 65 deviate clearly from a helical curve. In (b) a segment of residues 153–172 in a protein (pdbid 1MHY) is assigned as a single *α*-helix by dssp while our algorithm divides it into four helices: 3_10_-helix(154–156, yellow)–*α*-helix(157–163, green)–3_10_-helix(164–166, purple)–*α*-helix(167–171, green). However, a careful examination of the hydrogen bond energies for these residues in fact suggests that they could also be assigned to a 3_10_-helix even by the dssp standard (S2 Fig).

The differences in assignment exist not only in the number of residues or helices been assigned but also in the details of assignment such as the division of a single helix by one program into several helices by another, helix type swap and difference in helix termination at its C-terminus. Specifically our algorithm may divide a single helix by either dssp or stride into several helices of possible different types (Table 5, Figs 4 and 5). Furthermore, an *α*-helix or a part of an *α*-helix assigned by either dssp or stride may be assigned as either a 3_10_ or a *π*-helix or vice verse (Figs 4 and 5). Such division happens when the energy difference between the three pairs of residues, (*i*, *i* + 3), (*i*, *i* + 4) and (*i*, *i* + 5), is relatively small (S2 Fig). A hydrogen bond-based algorithm may have difficulty making a proper helix type assignment because of the error in the computed hydrogen bond energy. As shown in Fig 2b and Fig 4a, some of the *α*-helices assigned by dssp have large bends in the middle. Such helices are absent in our assignment because the residues in such highly-bended positions must have their local axis angle *a* ≥ *a*_*max*_ where *a*_*max*_ = 40.0° (Fig 2a). Less frequently, for some continuous backbone segments dssp may divide them into two helices but our algorithm assigns it as a single one (Fig 4b). Two examples shown in Fig 5 illustrate that (1) for some dssp assigned *α*-helices our algorithm tends to assign a part of them as a 3_10_ helix, and (2) the difference in the assignment of the C-terminal regions of helices. The division of a single continuous *α*-helix assigned by the previous programs into several different types of helices though less intuitive at the first sight is in fact a more accurate description of a protein structure and as detailed late should be useful for the discovery of structure-function relationship in proteins.

Though *r*, *p*, *t*, Δ distributions for the five groups (H, G, I, E and R) have almost no overlap (Fig 1j–1l), the largest peaks in the *r*, *p*, *t* distributions for the three helix types do overlap with the minor peaks in the *r*, *p*, *t* distributions for the residues in group R (peak B and C of Fig 1p, peak D of Fig 1q and peak F of Fig 1r). These overlaps may make it difficult for our algorithm (likely also for the previous programs) to distinguish a helix from a segment of protein backbone that is typically assigned as either a loop or a turn. To reduce the possibility of mistaking a turn or a loop as a helix, a term $\frac{\Delta_{i}^{2}}{2\sigma_{\Delta }^{2}}$ is added in the helix score (Eq 2) to guarantee that the helical curve not only fits optimally to a quadruple of C_*α*_s locally but also fits sub-optimally to all the C_*α*_s of an entire helix. Similarly the C_*α*_ RMSD *δ* is used as a restraint in 3_10_-helix assignment to distinguish it from a turn or loop. The largest discrepancy in assignment by different programs is in the C-terminal region of a helix because of the structural similarity between the C-terminal region of a helix and a turn or loop. To improve the accuracy of the C-terminal assignment and to have consistent criteria for the termination of a helix we have used a C_*α*_ RMSD threshold (*δ*_*G*_) for the termination of a 3_10_ helix. In addition to the two thresholds *h*_*max*_ = 160.0 and *a*_*max*_ = 40.0° used for the merge of two adjacent *α*-helices and the termination of an *α*-helix, we also include a C_*α*_ RMSD threshold *δ*_*max*_ = 0.3 to make sure that the part of a helix to be merged does not deviate largely from a genuine helical curve.

In summary, one advantage of our algorithm is better assignment accuracy and consistency because it is required by our problem formulation and algorithm that all the helices must fit reasonably well to a helical curve. In contrast, for dssp and other hydrogen bond-based programs as long as there exists the characteristic hydrogen bonding pattern for a helix type the participating residues are assigned to that particular helix type. Different hydrogen bonding patterns are distinguished by the relative hydrogen bonding strength between the three pairs of residues (*i*, *i* + 3), (*i*, *i* + 4) and (*i*, *i* + 5). In the twilight region where the difference in strength is small, the hydrogen bond-based programs have difficulty making correct type assignment. Similarly for the geometrical restraint-based programs the error and variation in the input restraints reduce their assignment accuracy and consistency.

### 4.3 A clustering analysis of the assigned helices

**Fig 6:**
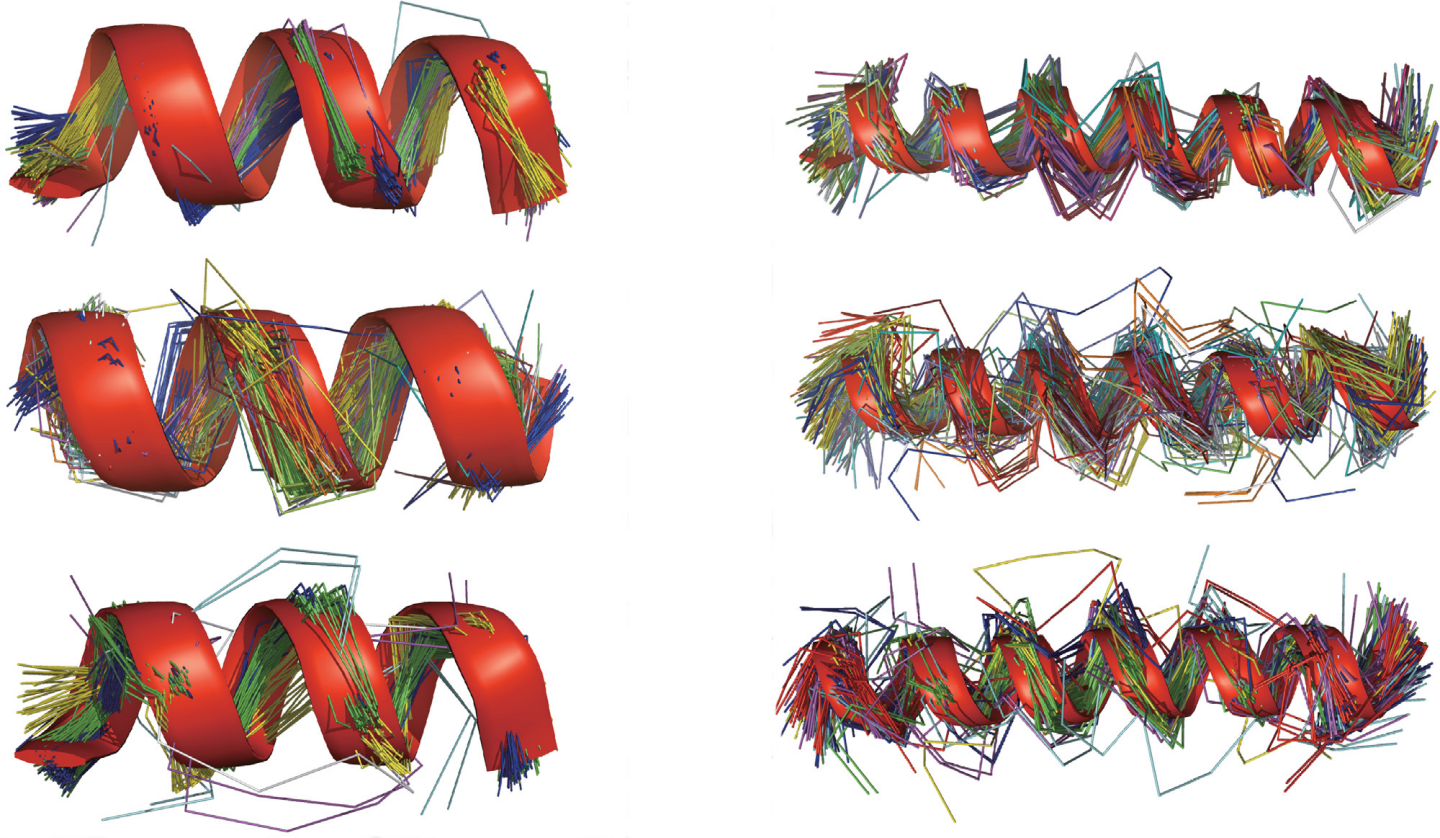
The clusters of *α*–helices by our algorithm, dssp and p-sea. The sets of helices in the left have a length of 12 residues while the sets in the right 24 residues. The 12-residue set (11,756 helices) and 24-residue set (1,211 helices) by our algorithm are classified respectively into 12 and 17 clusters. The dssp assigned 12-residue set (12,631 helices) and 24-residue set (1,285 helices) are classified respectively into 21 and 35 clusters while the p-sea assigned 12-residue set (5,306 helices) and 24-residue set (574 helices) are classified respectively into 10 and 24 clusters. The clusters are produced using our geometric clustering algorithm [22]. The RMSD threshold for clustering is 1.5Å.

One key feature that distinguishes our algorithm from the previous programs is that the helices assigned by our algorithm must fit to a helical curve reasonably well and thus are expected to be structurally uniform. To confirm the uniformity we have classified some sets of the helices assigned by our algorithm, dssp and p-sea on ℕ. The clustering is performed on sets of helices having the same length. Six sets of clusters on *α*-helices are shown in Fig 6. The first three sets of helices have a length of 12 residues and the second three sets a length of 24 residues. In general, the number of clusters in a set with the same helix length assigned by our algorithm is about the half of the number of clusters in the set by dssp. Though the total numbers of clusters by our program and p-sea are similar, p-sea is able to assign only a half of the helices with the same length. As shown in Fig 6, it is obvious that the structures of the helices assigned by p-sea are less uniform than those by our algorithm as is evidenced by the appearance in the former of several clusters consisting of only outliers. Please see S3 Fig of the SI for the comparisons of the clusters on four sets of 3_10_ and *π*-helices assigned respectively by our algorithm and dssp. In summary, the helices assigned by our algorithm are structurally more uniform than those by the previous algorithms.

### 4.4 The correlation between *π*-helices and protein-ligand binding sites

**Fig 7:**
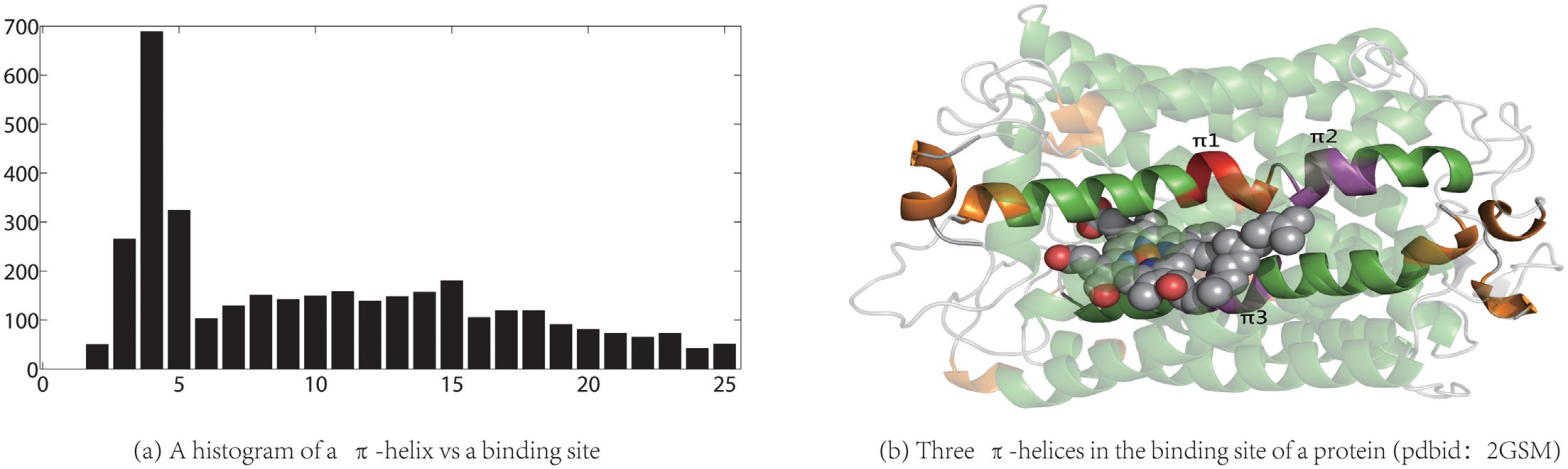
A histogram of a *π*-helix vs a protein-ligand binding site. Figure (a) shows a histogram of the *π*-helix assigned by our algorithm vs a protein-ligand site. The x-axis is the distance (in Å) between the ligand and a *π*-helix while the y-axis is the number of *π*-helices. Figure (b) shows an example illustrating the difference in *π*-helix assignment by our algorithm and dssp. Our algorithm assigns three *π*-helices labeled as *π*_1_, *π*_2_ and *π*_3_, all of them are in the ligand binding site but dssp fails to assign *π*_1_ though it is able to identify both *π*_2_ and *π*_3_. In this figure, *α*-helices are in green, 3_10_-helices are in orange and the ligand is shown as spheres.

Our algorithm is able to assign a helix to a particular type. As detailed above for *α*-helices, the agreement between our algorithm and either dssp or stride is excellent. However, for both 3_10_ and *π* helices the agreements are between 56.9% and 74.1%. An interesting application to structure-function relationship [26] is to see whether there exist any correlation between the location of a *π*-helix and a protein-ligand binding site. Out of the 25,806 protein structures in ℕ our algorithm has assigned 6,600 *π*-helices from 4,329 protein structures, 3,811 of them have a bound ligand. We compute the number of *π*-helices that is within a certain distance range of the bound ligand. The distance between a ligand and a *π*-helix is defined as the shortest distance between any ligand atom and any protein atom that belongs to the *π*-helix. As shown in Fig 7a there exists a strong correlation between the location of a *π*-helix and a protein-ligand binding site: 38.6 percent of all the *π*-helices assigned by our algorithm are less than 6.0Å away from a ligand binding site. Furthermore, as shown in Fig 7b, compared with the program dssp our algorithm is able to assign more of such *π*-helices. Such correlation should be helpful for the discovery of structure-function relationship in proteins.

## 5 Conclusion

We have divided the protein helix assignment problem into a minimization problem and a restraint satisfaction problem and developed an assignment algorithm that follows rigorously the geometry of helix. The application of the algorithm to the set of protein structures available in the current version of the PDB and the detailed comparisons of assignments made by the algorithm and the nine previous programs prove that our algorithm is able to assign more accurately not only *α*-helices but also 3_10_ and *π* helices as well as the left-handed helices. The clustering analyses of the helices assigned by our algorithm and the previous programs confirm that the helices from our algorithm are structurally more uniform than those by the previous programs. The accurately assigned helices and the clusters as well as the common structural features shared by all the helices in a cluster should be particularly useful for protein structure classification and prediction as well as secondary structure prediction. The accurate assignment of a helix to a particular type should be helpful for the discovery of structure and function relationship in proteins.

## Supporting Information

S1 Fig The distributions of C_*α*_ RMSD *δ* and helix axis angle of the residues in the C-terminal regions of dssp assigned helices. The x-axis is the helix axis angle in degree, the y-axis is the C_*α*_ RMSD *δ* in Å. The region inside the rectangle defined by the two intervals, [0.0°,40.0°] for the axis angle and [0.0,0.1Å] for C_*α*_ RMSD *δ*, includes 83.5% of all the 29,014 data points computed on set 𝕊. What shown here is the data for residue *i* for the two C-terminal residues *i*, *i* + 1 of a dssp-assigned helix.
(EPS)

S2 Fig The hydrogen bond energy used by dssp for the helix assignment. Figure S2 is extracted from the dssp assignment for the protein 1MHY (pdbid). This example illustrates that in the twilight region where the differences in hydrogen bond strength are small, a hydrogen bond-based program such as dssp may have difficulty making correct helix type assignment. The three red rectangles indicate donor interaction while the three blue ones acceptor interaction. The data is from dssp for the protein 1MHY (pdbid) the same protein as shown in Fig 5b of the main paper. The program dssp assigns all the residues from 152 to 172 as a single *α*-helix while our algorithm assigns the segment from 154 to 156 and the segment from 164 to 166 as two different 3_10_-helices. A careful examination of the hydrogen bond energies for the residues in the six rectangles in fact suggests that they may also be assigned to a 3_10_-helix even by the dssp standard.
(EPS)

S3 Fig A clustering analysis of 3_10_ and *π*-helices. The figure shows the clusters generated by the geometric clustering algorithm on sets of the 3_10_ and *π*-helices assigned respectively by our algorithm and dssp. The two sets of 3_10_ helices in the left (a) have a length of 4 residues while the two sets of *π*-helices in the right (b) have 5 residues. The 4-residue set (8,861 3_10_-helices) and 5-residue set (4,554 *π*-helices) by our algorithm are classified respectively into 4 and 7 clusters (the two upper figures). The dssp assigned 4-residue set (8,563 3_10_-helices) and 5-residue set (5,365 *π*-helices) are classified respectively into 29 and 25 clusters (the two lower figures). The clusters are produced using our geometric clustering algorithm [22]. The RMSD thresholds for the clustering of 3_10_-helices and *π*-helices are respectively 0.3Å and 0.75Å.
(EPS)
